# Humanitarian–development nexus approach to health systems strengthening in Sudan—a policy analysis

**DOI:** 10.3389/fpubh.2025.1579825

**Published:** 2025-06-18

**Authors:** Huzeifa Jabir Idris Aweesha, Anna-Karin Hurtig, Anni-Maria Pulkki-Brännström, Miguel San Sebastián

**Affiliations:** Department of Epidemiology and Global Health, Faculty of Medicine, Umeå University, Umeå, Sweden

**Keywords:** humanitarian, development, nexus, Sudan, policy, health, system, partnership

## Abstract

**Background:**

With increasing conflict, fragility, and emergencies in many countries, health systems are being frequently weakened and require support and strengthening. To ensure the provision of lifesaving interventions while improving national health systems, the humanitarian–development nexus presents a policy solution to bridge the divide between humanitarian and development actors. Sudan represents an interesting case of the nexus of adoption in the context of protracted emergencies, a volatile political scene, and complex economic and partnership dynamics. This study aimed to explore the understanding and adoption process of the nexus approach to health systems strengthening in Sudan.

**Methods:**

We conducted a policy analysis based on seven qualitative interviews conducted in 2022 with informants from varying humanitarian and development entities.

**Results:**

Our findings revealed uncertainty surrounding the meaning and practicalities of the nexus, despite a consensus on its importance. At the same time, the introduction process was driven by global partners, the Government of Sudan’s adoption of the nexus, and the presence of relevant coordination mechanisms within the health sector, which facilitated the advancement of the nexus. However, the humanitarian and development actors expressed conflicting values and disagreements about the use of the financial management, procurement, and information components of the national health system.

**Conclusion:**

Sudan’s health partners have implemented various nexus-based practices to strengthen national health system capacities. However, the nexus approach has faced challenges due to conceptual ambiguities and inconsistent implementation. We call for the advancement of guidance and dialog on the approach, emphasizing the importance of ownership, coordination, and flexibility, with the belief that the humanitarian–development divide can be further bridged.

## 1 Introduction

The Global Humanitarian Overview 2019 (1) indicated that over 2 billion people globally live in Fragile and Conflict-Affected States (FCAS), primarily within low- and middle-income countries, requiring assistance with vital services and restoration of their national systems and capacities. The living conditions within those settings are complicated by emergencies such as infectious disease outbreaks, in addition to the already high burden of injuries and illnesses from weak preventive services or difficulty in accessing vital services (2). The conflicts and fragilities within these settings, which demand more than the system’s capacities, and the subsequent repeated health emergencies contribute to disrupting the health systems (3, 4). Despite increasing Official Development Assistance (ODA) targeted at the health systems and health services, the challenges persist and are complicated by the proliferation of uncoordinated global initiatives and actors (5), as well as the tendency of humanitarian NGOs to undermine local health systems strengthening by delivering health services independently (6). Following the adoption of Universal Health Coverage (UHC) as the primary health target within the Sustainable Development Goals, the importance of investing in health systems strengthening to achieve UHC has been more notably recognized (7, 8). Humanitarian organizations were reported to be resistant to investing in strengthening the public health system’s capacity and to filling service gaps (9).

In their review of systematic reviews on health systems strengthening in FCAS, Bogale et al. (2) indicated a consensus on the importance of coordinated and integrated response by all actors to maintain the health systems based on the context and stage of the crisis. Olu et al. (10) underscored the importance of addressing the humanitarian–development divide and using health as an enabler for peace as a crucial approach to addressing health system challenges in FCAS to attain UHC and other health targets. Similarly, the World Health Organization (WHO) argues that improving health and advancing health systems necessitate a coordinated approach of the nexus among humanitarian, development, and peace actors that responds to immediate needs while rebuilding the health system and addressing risks and factors to prevent future emergencies, conflicts, and fragility (11). Accordingly, the WHO developed a guide for implementing the nexus approach in the health sector. The guide was intended to advance the nexus as a policy solution to bridge the divide among these actors towards improving health systems and achieving the health targets.

The humanitarian–development nexus (hereafter referred to as “the nexus”) is a term that has been widely used among global actors on the frontlines of humanitarian work and development cooperation (12). Strand defines the humanitarian–development nexus as the transition or overlap between the delivery of humanitarian assistance and the provision of long-term development aid (13). The concept is argued to be an old idea that has evolved through various initiatives and combines their objectives (11, 14). The nexus brings aspects from the Linking Relief, Rehabilitation, and Development approach from the 1980s and 1990s where there was a drive from donors, specifically, the European Union (EU), to link “short-term relief” with “longer-term development” to create synergies and provide a sustainable response; this was however criticized for being a linear sequence where rehabilitation had to follow relief and then development came last (15). This was not practical within the context of protracted emergencies; hence, a continuum approach of simultaneous complementary action, and later the “building resilience” strategy aiming to combat fragility, prevent conflicts, prepare for disasters, and reduce risks was advocated by the early 2000s.

The new millennium brought up the discussions of the Development Goals, with more focus first on development aid effectiveness and then on effective development cooperation principles, which emphasized the ownership and leadership role of recipient countries (usually targeted due to their weaknesses) and hence directed more work towards building national systems, institutions, and capacities. Moreover, the Sustainable Development Goals (SDGs) (16) emphasized “leaving no one behind,” which entails targeting the poorest and the most vulnerable, who are also often the targets of humanitarian action, leveraging new thinking on how to break the cycle of emergency needs. This was further advanced when the World Humanitarian Summit (17) called to transcend the humanitarian–development divide through a “grand bargain” that addresses the drivers and risks of conflict. This was translated from the United Nations Secretary-General’s (UNSG) address to the UN General Assembly in 2016, where he launched the humanitarian–development–peace nexus (18). Within this triple nexus, he urged active engagement for humanitarian response, sustainable development, and peace. The modalities of this engagement were further detailed in the United Nations’ (UN’s) New Way of Working approach (19), where discussions about collective outcomes emerged. Collective outcomes were defined as “concrete and measurable results that humanitarian, development and peace actors want to achieve jointly over 3–5 years to reduce people’s needs, risks, and vulnerabilities and increase their resilience” (20).

Sudan represents a case of prolonged and multiple conflicts, instability, economic regression, protracted varying emergencies, and complex international relations (21, 22). Following the longest war in Africa’s recent history, a peace agreement in 2005 and a referendum led to the separation and establishment of the new South Sudan in 2011. Multiple further conflicts erupted in various parts of the country during the following years. Ever since Sudan suffered a revenue reduction due to the loss of the resource-rich South, international donors have provided less support due to the challenging relations with the regime (23). Sudan’s political and economic context has undergone significant changes, from being under sanctions by the United States and other global powers until 2018, when a revolution overthrew the regime with post-revolution openness and an influx of partners (24), which was subsequently disrupted by a coup that led to the overthrow of civilian rule in 2021 (25).

Multiple big donors and international agencies selected Sudan to be among the pilot countries for the nexus approach. First, the EU selected Sudan in 2017 among five other pilot countries for the nexus (including Chad, Nigeria, Iraq, Myanmar, and Uganda), and then the World Bank and UN started piloting the New Ways of Working, in 2018, jointly in Sudan, Somalia, Cameroon, the Central African Republic, Guinea Bissau, Pakistan, and Yemen (26). In 2017, a peer-to-peer workshop on strengthening collaboration by adopting the nexus was held in Khartoum with the participation of prominent donors, the UN Country Team, and other global implementing agencies. This workshop stated that the country’s context was favorable and necessitated nexus implementation and financing, specifically highlighting the commendable health sector for having a high-level coordination mechanism that could oversee and facilitate the nexus (27).

There is a lack of studies that examine the nexus adoption process in the health sector to draw context-specific lessons, especially for such fragile and conflict-affected settings. Following the contextual changes since the nexus approach was introduced in Sudan in 2017, it would be valuable to draw lessons from this case on adopting and implementing the approach in Sudan’s health sector. This study aimed to explore the understanding, conceptualization, and adoption process of the nexus approach to health systems strengthening in Sudan.

This paper focuses on the humanitarian–development nexus within the Sudan health sector, not the triple nexus. It does not include peace actors, as they were not visible in the country’s health sector.

## 2 Methods

A policy analysis was conducted using the policy triangle framework (28) to facilitate the exploration and understanding of the conceptualization process, practices, challenges faced, and opportunities foreseen by partners involved in the nexus adoption and implementation of the nexus within Sudan’s health sector.

### 2.1 The setting: Sudan health sector humanitarian and development partnerships scene

The health sector in Sudan includes a range of humanitarian and development partners. On the humanitarian side, the health cluster was established in 2009 as the primary mechanism to coordinate the activities of humanitarian partners, comprising approximately 24 national NGOs, 23 INGOs, 8 United Nations agencies, 5 donors, and the government, represented by the Ministry of Health’s relevant departments. The health sector development partnership scene is more limited in the number of international agencies and NGOs. Donors for the development of the health system portfolio include multilateral donors like the Global Fund to Fight AIDS, Tuberculosis and Malaria (GFATM), and the Global Alliance for Vaccines (GAVI), and bilateral donors like Japan, Italy, and development banks (Islamic Development Bank, African Development Bank). Donors, such as the EU and World Bank, have moved in the past 5 years to provide development assistance in addition to their usual humanitarian aid. The leading UN agencies supporting the health sector are the WHO, UNICEF, and UNFPA. Few INGOs work in health systems strengthening. The sector includes multiple government ministries, funds, and councils, all overseen by the Ministry of Health (22).

**Figure 1:**
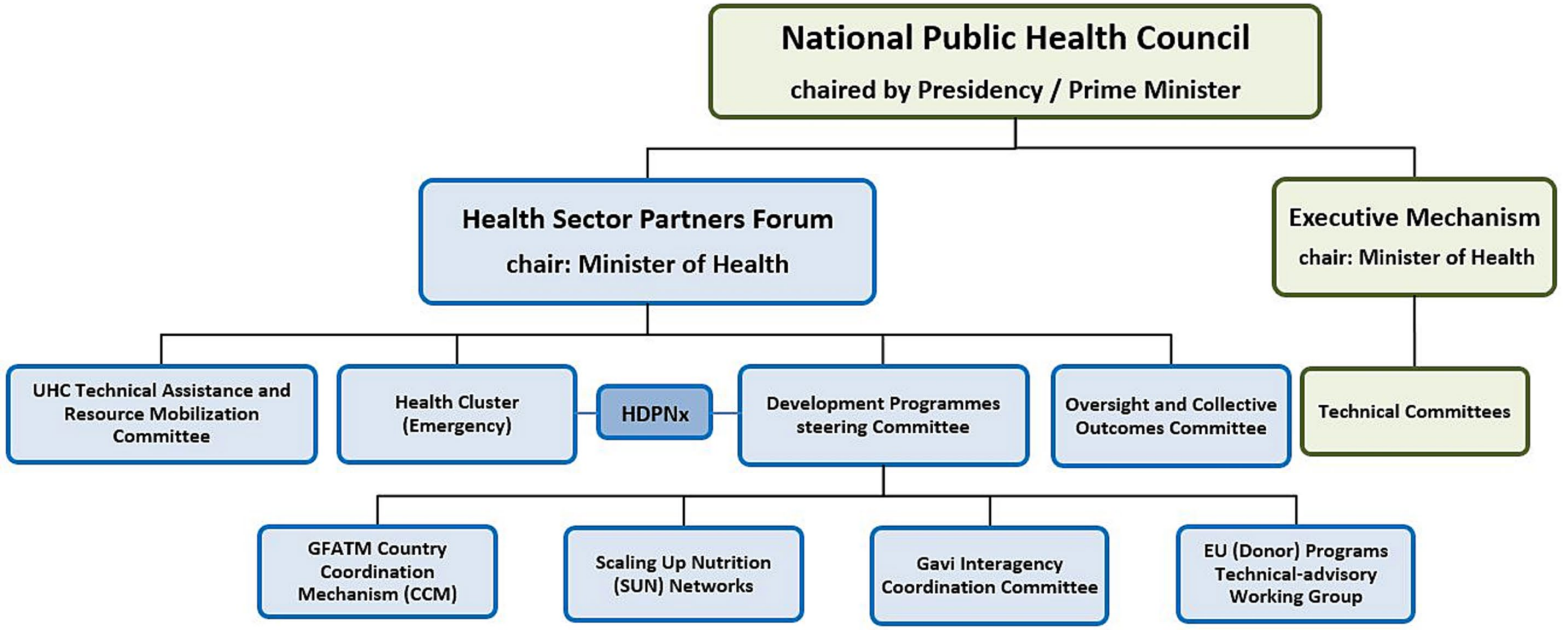
Sudan health sector coordination structures (adapted from the NHRR-SP 2022–2024).

Over the last decade, the health sector has suffered from fragmentation and disconnection between humanitarian and development partners, as well as the coexistence of multiple coordination mechanisms on the development side. Thus, the Health Sector Partners Forum (HSPF) was established in December 2016 as a coordination mechanism to ensure policy coherence and reduce fragmentation. It was later further tasked with advancing the nexus work through the consideration of the health cluster as one of its committees, similar to its parallel development programs steering committee (22). Figure 1 illustrates the coordination structures from the Sudan National Health Sector Recovery and Reform Strategic Plan (NHRR-SP) 2022–2024. The figure highlights how the nexus approach was introduced through joint meetings of the emergency cluster with the development programs steering committee, and further at the policy level in the overall Forum meetings, which included both humanitarian and development partners.

### 2.2 Data collection and participants

A semi-structured interview guide was developed by the researchers based on the study objective (see Supplementary material). The interview guide addressed issues related to the nexus understanding, introduction, and implementation process, as well as its implementation progress, challenges, and opportunities. While exploring these aspects, the policy aspects of the content/concept, actors, process and context were kept in mind and explored. Seven individual interviews were conducted, during April–May 2022, with respondents including leading personnel (directors and managers at national and sub-national levels) in health partner institutions of relevance to the nexus from the constituencies of Government, donors, UN agencies, and international non-governmental organizations (NGOs), from both the humanitarian and development domains. The initial three participants were purposefully selected by the first author based on their involvement with the nexus topic, and snowball sampling was used to identify the remaining respondents. The interviews were conducted via online Zoom calls, as Zoom was widely used for partner meetings in the Sudanese health sector following coronavirus disease 2019 (COVID-19). All respondents confirmed their preference for it, and the process of using it went smoothly, as all respondents, even those in the field, were of high rank with good connectivity arrangements. Interviews were recorded with respondents’ consent. Relevant documents referred to by respondents during the interviews were reviewed, and the minutes of a coordination meeting and observations were used to supplement the data from the interviews.

The interview guide was frequently amended during the study process. After each interview, revision of notes and a review of memos led to making necessary changes, keeping the backbone of the guide, while prospectively modifying or adding to address arising concerns. All interviews were conducted in English as all respondents were fluent in it.

### 2.3 Data analysis

A reflexive thematic analysis approach was adopted following Braun and Clarke’s guidelines (29). The recorded interviews were first transcribed verbatim, revised by listening back to recordings and rereading the transcripts to ensure accuracy and familiarize more with the data. Notes and short memos were incorporated into this process. Using the OpenCode 4.03 program software ICT Services and System Development and Department of Epidemiology and Global Health at Umea University, Sweden (30), line-by-line coding of the transcripts was performed inductively. Codes were compared and reflected on against the study aims and memos, and subsequently grouped into initial sub-themes. This was fostered by rounds of revising the groupings (initial sub-themes) and the codes’ fit on each and their relevance to the data. Then, examining patterns across sub-themes helped formulate themes, aided by memos and notes. The themes were further revised and organized using the policy triangle framework (28).

The policy triangle framework is grounded in a political economy perspective, considering not only the policy content but also the actors and their relationships, the context at play, and the process of the policy introduction and implementation. It is of good relevance to our study as we investigated a policy where the policy content was defined by the nexus understanding, and the policy actors—the health partners from both humanitarian and development spheres—had complex relations, power, and interests. The process of the policy was outlined by the conceptualization, adoption practices, and challenges, while the context within which this nexus policy was introduced and implemented is the surrounding sphere of the politics, economy, and society in Sudan.

### 2.4 Ethical considerations

Ethical clearance was obtained from Sudan’s Ministry of Health Research Ethical Committee in February 2021. Information about the study was shared with targeted respondents. Written informed consent was obtained prior to the interview and complemented by oral informed consent at the beginning of each interview. The respondents were assured of data confidentiality and requested to permit the recording of the interviews, and they all accepted. Data, including interview recordings and transcripts, are being kept securely, and the presented results do not include details that could reveal participants’ identities.

## 3 Results

**Figure 2:**
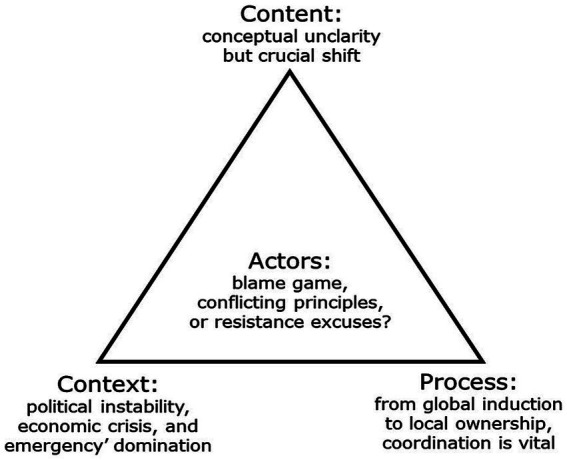
Policy triangle adaptation of the study results.

The results are organized using the four elements of the policy triangle framework (Figure 2). The main findings reveal that the nexus as a policy concept suffered from conceptual ambiguity, despite a consensus on its criticality in this context. The process was initiated by global actors, but crucial local ownership was established, and coordination was vital for progress in adopting the approach. The actors exchanged blame, with claims of conflicting principles and excuses for resistance. Political instability, economic crisis, and health emergencies dominated the policy context.

### 3.1 The content: conceptual unclarity but crucial shift

The respondents expressed an overall lack of clarity of the inherent nexus concept’s meaning, with various actors claiming the title for their various approaches to work.

“I saw four approaches toward [the nexus] in Sudan; Darfur development strategy revival that started in 2013 argued moving from humanitarian phase toward development phase, then the European Commission was operationalizing the systems resilience as nexus, we had the United Nations, which was defining the so-called collective outcomes, and also the World Bank with the cash transfer claiming to move away from humanitarian work. So, there was no one and only nexus concept in Sudan.” (Respondent 2 – Donor)

This was further commented on by several respondents who described the necessary shifts the nexus brought in terms of long-term thinking when working on immediate emergency relief, still admitting various definitions.

“There are different definitions but for me, the Nexus is an approach whereby humanitarian and development and sometimes peace actors who are working to address the immediate needs of communities, at the same time consider and act on long-term needs entailing rebuilding or strengthening systems, local capacities, particularly concerning preventing and responding to emergencies, as well as recovering from emergencies and tackling the root causes to break the cycle of recurrence.” (Respondent 4 – INGO)

One application of this thinking in the health sector was, as mentioned by some participants, to have innovative solutions to ensure sustainable health services for facilities run through humanitarian actors and emergency funds. This included working and providing services through the national systems to serve beneficiaries, specifically those under humanitarian conditions and supported by humanitarian partners. For instance, it was mentioned that the National Health Insurance Fund (NHIF) could be contracted to cover the population under its umbrella, where people could be connected to already existing clinics rather than establishing new ones through humanitarian funds, and similarly with other systems. This was seen as a collaboration opportunity to enable continuity of vital functions in the health sector in line with the global health target of UHC, and to ensure protection from health hazards at the same time.

“Reaching UHC by 2030 and its most important component the interconnectedness between UHC and health security, is simply talking about the nexus. Adopting the nexus thinking, humanitarian partners can subscribe their target population to be covered by the NHIF …. to procure through the National Medical Supplies Fund and to make use of the District Health Information System II for all information reporting.” (Respondent 1 – UN agency)

### 3.2 The process: from global induction to local ownership, coordination is vital

The introduction and implementation process of the nexus in Sudan has been significantly influenced by global actors and agendas that have actively driven the concept in the health sector. Following the World Humanitarian Summit’s adoption of the approach, it was quickly brought to Sudan in 2017 by the donors and UN agencies, excluding the Government from the initial deliberations.

“Nexus workshop was held in 2017 in Khartoum between [development Donors] and [humanitarian donors]. UN was invited but we were not engaging with the Government. It was mainly the so-called humanitarian actors and so-called development actors talking to each other.” (Respondent 2 – Donor)

Sudan’s active participation in global development cooperation discussions facilitated the adoption of the nexus, among other international practices, for local use as outlined by foreign partners.

“Sudan was a signatory for the IHP+ compact and all relevant global cooperation initiatives. That allowed and provided a bigger ground to roll out new approaches that kept coming up. So, with those participations in global initiatives, the nexus uptake was much easier for us, in terms of bringing up such approach to Sudan.” (Respondent 1 – UN agency)

Over time, it became clear that local ownership and actors were crucial to the approach’s success. The same respondent above went on to state the importance of translating the nexus-oriented actions by the government into practical, on-the-ground initiatives at lower levels for them to succeed.

The Government of Sudan took a positive stance on the nexus, adopting it as a strategic shift necessary within the country’s challenging, emergency-dominated context to ensure the sustainability of health services and hence address the root causes of the emergencies.

“The nexus is part of the strategic directions for the Federal Ministry of Health … it would support the advancement of the health system in the context of protracted emergencies. The investment in emergency response is very high, yet we keep having these emergencies because there is no sustainability impeded.” (Respondent 5 – Government)

It was even seen as a necessity that champions from all government ministries and agencies would be needed to ensure the success of the rollout and adoption of the nexus, not just at the Ministry of Health. These local champions were considered necessary due to the resistance the nexus faced and the knowledge gap among many crucial government actors.

“Still there is a level of resistance due to a lack of understanding. Until nexus is discussed in all the fora and ministries through dedicated personnel the knowledge will not increase, and nexus will not be fully embraced.” (Respondent 1 – UN agency)

On top of the above, the respondents declared the crucial role of coordination forums for the nexus. They strongly attested that the Health Partners Forum, the coordination umbrella that brings together humanitarian and development health partners, played a critical role in advancing nexus thinking within the health sector, as it had initiated nexus discussions before any other sector did.

“At the HSPF, as partners working on both humanitarian and development spheres, together we discussed how to learn from each other and spread the nexus thinking further. That contributed to our understanding by making it the theme for a Forum meeting in 2017, and for the first time it was discussed by any sector.” (Respondent 1 – UN agency)

Discussing the nexus at this level positively influenced the effort to institutionalize the approach, becoming one of the strategic directions within the subsequent National Health Policy (NHP).

“Picking the nexus at the highest level, bringing it to institutionalization through the NHP. The forum approved the policy, so the discussion transformed into operationalization… This is where the critical role of the HSPF comes in.” (Respondent 1 – UN agency)

The health sector even had a more advanced oversight structure for governance purposes, namely, the National Public Health Council (NPHC), which led deliberations on policies and ensured the Government’s commitment and harmonized action on the nexus. The presence of this structure translated into fruitful examples of policy documents and agreements.

“Look at the health in all policies (HiAPs), have you heard about Nutrition or WASH or refugees in all policies? … only one sector [health] has MoUs signed with 18 ministries. Through the HiAPs, if I have an issue with animal-transmitted diseases I go to the Ministry of Animal Resources, if I have an issue with technologies not getting clearance, I can go to the ministry of trade and tell them this is a TRIPS issue.” (Respondent 1 – UN agency)

Despite all this praise of the health sector coordination mechanisms, the relationship between the humanitarian health cluster and the Health Partners Forum was not well established, and not all humanitarian actors were involved in the nexus discussions via the forum.

“While the [Ministry of Health] said the cluster is a working group of the partners' forum, I didn’t have the impression that the humanitarians saw it like that. They saw it as a form of NGOs talking to NGOs and didn’t have the commitment to see it as a working group of a broader government-led coordination mechanism.” (Respondent 2 – Donor)

After the 2019 revolution and the subsequent instability, these mechanisms became less active.

“The HSPF and the fora that we used to coordinate with the other sectors, due to the instability, have not been active anymore for some time.” (Respondent 5 – Government)

The respondents also reflected on the lack of active coordination structures in other sectors (such as education, nutrition, and other service sectors). This was linked to the resulting domination of emergency interventions and short-term planning in different sectors, delaying progress toward the nexus.

“The coordination structures that exist in the health sector do not exist in any other sector, so their speed of implementation was slower because they are rediverting towards the emergency side like we see the nutrition people are again talking about emergency grants. Structures must be developed in all sectors.” (Respondent 1 – UN agency)

Many respondents asserted that in other sectors, nexus institutions had not been developed, and coordination structures were in their infancy. The explanation behind this was attributed to working in silos which is against the collective thinking manner of the nexus. A way to address this was proposed by some participants.

“Other sectors need to be better organized, and we need to talk together at some overarching level and have harmony to be able to achieve our goals.” (Respondent 7 – Government)

### 3.3 The actors: blame game, conflicting principles, or resistance excuses?

Contradictory views and blame exchanges were raised by both humanitarian and development organizations regarding the progress in adopting the nexus approach in Sudan’s health sector. Development partners believed that humanitarian actors were abusing the nexus concept to secure funds from donors.

“There was a lot of talk about protracted crisis, it was driven in my opinion by humanitarians as they see a crisis in countries like Sudan, they don’t have the money to continue financing but want the development partners to take over what they have been doing and to fund the kind of work that they have been doing with development money.” (Respondent 2 – Donor)

The respondent described humanitarians as “actors” in opposition to development “partners,” relating this difference to the existence of effective cooperation agreements in the development world that entail active partnership efforts beyond the implementation of interventions. He expressed a belief that humanitarian and development principles do not mix well.

“Humanitarian and development principles are different and mutually exclusive … you cannot be independent and aligned at the same time.” (Respondent 2 – Donor)

Engaging with local institutions and utilizing national systems was challenging for humanitarians as expressed by the development partners. This was complicated by poor knowledge and communication between humanitarians and the local institutions, and further by the regulations from humanitarian donors.

“Humanitarians for the first time realized that they have to work with Ministry of Health and with the National Health Insurance Fund, even though they didn’t know what to do with them, and with the National Medical Supplies Fund for medical supplies, yet [some donors] do not allow them to procure locally.” (Respondent 1 – UN agency)

On the other hand, humanitarians believed that the country’s systems and capacities were weak and that the development work was in its infancy, requiring considerable effort to be done before such a shift.

“Development National Systems are weak. Look at DHIS II as an example, you will find it, after 10 years of support, very weak, not producing enough data, relying on external fund, not supported internally by the authorities but somehow supported by development actors still not producing the needed data at real-time.” (Respondent 3 - Humanitarian)

They reflected on the continuity of humanitarian funds despite all the recent political changes, unlike development grants. They argued that the nexus of progress depended on the development side further advancing the health system capacities.

“As humanitarians, our duty continues to save lives and for that, our interventions continue despite any politics. But to apply nexus for sustainability, we need to wait for the other side to start building towards our way as well so that we can meet somewhere in the middle.” (Respondent 3 – Humanitarian)

The humanitarian partners further argued that although the regulations limited the use of some national systems and procedures, the results were the same as they aimed to save lives; therefore, the choice of which systems to use was only a matter of efficiency.

“I cannot agree much on [national systems use as necessity] because if we cross match what emergency partners do, and what development do with national systems … it is the same. For example, an agency can support the EWARN community surveillance system. On the development side, they have the same approach, but they are adopting different systems and both systems give the same result. It is just a matter of a system prevailing over another because it's more functional. In my view as long as my systems are able to function better than the development systems then I will keep using them.” (Respondent 3 – Humanitarian)

While some pilot projects attempted to utilize the country’s systems (such as procurement, financing, management, and information), the resistance from those working in humanitarian assistance was evident, as if they did not understand or were unwilling to implement such changes.

“After we transitioned health facilities to be supported by local authorities and insurance, there were staff who really could not understand why we were not continuing to pay incentives directly but through the insurance system, why going for insurance cards rather than the previous free services. Mindsets were difficult to change.” (Respondent 4 – INGO).

### 3.4 The context: political instability, economic crisis, and emergency domination

Sudan has been suffering from political instability and economic crises for the past decades. On top of this, the protracted emergencies with unstable funding that hindered sustainability made the whole nexus implementation a mission of shooting in the dark without enough clarity of where the country is starting from or where it is heading.

Emergencies have dominated the healthcare scene, with emergency actors and interventions, and projects that were predominantly short-term and funded. This has created a situation that challenges the sustainability of services, as the population in many areas affected by protracted emergencies is dependent on INGO-based short-term health projects.

“Humanitarian projects have a very short life span. That is a bottleneck. We get funding for 6 months to provide lifesaving interventions and then the services are interrupted, and the health services and people rely on this emergency repeated cycle.” (Respondent 6 – INGO)

For some participants, this concern with health services continuity under these humanitarian circumstances motivated the adoption of the nexus, ensuring humanitarian–development bridging with a focus on sustainability and development-oriented thinking that enabled resilient ways of working.

“We need sustainable investments even with protracted emergencies, and the nexus can facilitate such shift.” (Respondent 5 – Government)

The challenges of Sudan’s political instability and economic crisis presented a hurdle to progress on the nexus as a big policy to be adopted within a complex and challenging context. While deliberations on the nexus introduction in Sudan were initiated, these crises were direct challenges that complicated and limited the humanitarian–development cooperation efforts.

“Economic crisis is a major challenge. We were making progress in adopting the nexus, but this came as a big problem, and everything just stopped.” (Respondent 1 – UN agency)

“The projection on the point where we can say let's scale down humanitarian interventions and build up the other side {development systems} has been obscured after October 25th [2021 coup] as it got clear development donors will suspend their support. We were looking at the other bank of the river and it suddenly vanished.” (Respondent 3-Humanitarian)

The respondents expressed concerns that these challenges complicated the development side of the nexus further and threatened any cooperation efforts. They argued that this status needs to be resolved; otherwise, the continuation of the humanitarian emergency cycle would be imminent.

“How do you talk about development where there is no fuel, no dollars, and no subsidies for basic items.” (Respondent 1 – UN agency)

“In Sudan context now, I believe that we have a lot of disablers. Humanitarian actors exist, government exists with low capacity, but development actors had disappeared recently. So, we have a broken triangle.” (Respondent 3 – Humanitarian)

## 4 Discussion

The policy analysis has shown how the conceptual unclarity of the nexus, combined with its introduction through global actors rather than innate local interests, and the challenging context, have all negatively impacted the adoption and progress of the nexus in Sudan’s health sector. Nevertheless, the government’s acceptance of the approach, along with the existence of relevant coordination structures in the health sector, has contributed to some successes. The concept has gained familiarity among many partners, and several implementation endeavors are underway. Practices of contracting the NHIF to cover humanitarian target populations, procuring through the NMSF, using the DHIS II for reporting, and supporting existing health facilities rather than establishing new ones were listed as the main ways partners are working on the nexus approach to strengthen the national health system and capacities. Yet, this was expressed differently by different respondents. Some focused on the nexus as a way of improving the national system’s resilience, enabling it to respond and recover from emergencies. Others have seen it as a way of transitioning from the humanitarian phase into development, which humanitarians have found problematic due to the national system’s weaknesses. Overall, the relations between humanitarian and development partners remain complex and require transparent dialog and active intervention.

### 4.1 The conceptual unclarity—call for further guidance and dialog

Although the nexus builds on previous initiatives that evolved over decades, and despite the efforts to define the nexus, especially from the United Nations, the uncertainty on what it means and the conflicting variations of the way it is tackled were strikingly clear within this study. While most published papers and guide documents have focused on the definition of the nexus as being the action of “who” (humanitarian, development, and peace actors) and for “whom” (11, 12, 26), our study raised further concerns on “what” and “how.” There is a clear lack of consensus on what should be done and what applying the nexus entails, highlighting a guidance gap in its implementation. While WHO has a nexus implementation guide from the Eastern Mediterranean Regional Office (11), this guide does not address the concerns of the conceptual definition and detailed implementation procedures needed. Within this guide, the nexus is defined as any work where at least two of the three groups of actors work together to provide immediate life-saving assistance, strengthen or rebuild the national systems, institutions, and capacities, or address the drivers of emergencies. Although this guide attempted to address the triple nexus within this definition from an operational perspective, it ultimately described it as an act involving just any two groups of actors. The same UN agency has a separate position paper on building health systems resilience for UHC and health security (31) that only mentions the nexus 3 times and has no practical guidance points. The Universal Health Coverage 2030 partnership (UHC2030) strategic paper, which aims to guide advocacy and action on health systems for UHC and health security (32), also lacks any mention of the nexus, despite its clear relevance.

Furthermore, Sudan’s health sector partners signed a compact in 2014 detailing their cooperation commitments; however, the compact lacks any mention of the nexus specifically or any similar initiatives that bring humanitarian and development actors closer together and ensure their complementarity for sustainable impact and resilience. The compact has not undergone a comprehensive review process, despite multiple changes in national and global arenas, including the SDGs, the introduction of the nexus, and the revolution, which have led to subsequent changes in Sudan.

That being said, it might be true that having a unified way to implement the nexus is not feasible, nor the best choice, as explained by Joireman and Haddad (33) in their review study. Rather, advocating for experience-sharing and joint planning through continuous cooperative platforms would enrich the understanding and the variety of possible nexus practices.

Another concern that was noticeable in our study and the literature is the utilization of some terminologies relevant to the nexus understanding that have different meanings within the humanitarian and development spheres. The most pertinent example is the use of the term resilience, which for development actors relates to livelihoods and climate change in addition to the system’s capacities, while for humanitarian actors, resilience conveys the meaning of managing risks and increasing flexibility to withstand emergencies (34). Such conceptual differences increase the importance of discussion among the actors to foster mutual understanding.

Moreover, this study has demonstrated how the context within which the nexus is in interplay, as well as the interests of the key actors, particularly those with more influence, shape how the nexus is conceptualized and understood in different situations. During the post-2019 revolution transitional government period in Sudan, multiple actors, including the Government and donors, were pushing a move from humanitarian aid toward development cooperation, and this changed drastically following the 25 October 2021 coup, as development cooperation almost vanished since donors decided to freeze their support to Sudan (24). Later, after the war was declared on 15 April 2023, in the country, a reverse course was initiated, where some development grants under the nexus title are being repurposed for life-saving interventions (35). Due to this instability, Mohamed Nur et al. (36) in their practice report on a nexus project repurposing in Sudan, pointed out the importance of perceiving the nexus as a continuum rather than sequential in one direction. They further stressed the importance of flexibility in design and adaptability to such a volatile setting, and additionally pointed to the guidance gap.

### 4.2 Conceptualization process—coordination, ownership, and flexibility importance

The study reflected on an interesting process initially driven by the interests of international partners, while sidelining the national entities; however, the Government of Sudan reacted positively to the nexus and took steps to advance it. This was achieved through the conduct of Sudan Health Sector Partners Forum meetings to discuss the nexus and share experiences, and to further incorporate the nexus as one of the key health policy transformations. These actions by the Government of Sudan contributed to the success of nexus practices.

This positive uptake of the nexus, despite the initial lack of ownership and respect, shows that the Government of Sudan was generally flexible in committing to globally driven initiatives when responding to the real needs they faced. The nexus was an opportunity to advance development interventions, including health systems strengthening, within a volatile and emergency-dominated context. The global actors on the other side soon realized the importance of having national ownership and leadership for the initiative. This corresponds well to the principle of adaptive agents who react to the overall system’s needs and to each other, and accordingly self-organize within a complex system and context (37).

The role the coordination mechanism (Sudan Health Sector Partners Forum) played in the initial phase of nexus adoption in Sudan was praised, as it led to the emergence of practices reported here. This was further elaborated on in the Sudan nexus profile for health (38). The Forum also contributed to the inclusion of the nexus as a transformational shift in the Sudan National Health Recovery and Reform Policy 2021–2024. The absence of such coordination mechanisms in other sectors was identified as a primary cause of delay in nexus adoption. Future nexus implementation will benefit from revitalizing and enhancing the forum. We also recommend replicating the health sector coordination models in other sectors with comprehensive ownership and uptake of the nexus by the whole Government of Sudan.

### 4.3 The humanitarian–development divide: can it be avoided?

Our study highlighted the problematic division between humanitarian and development partners, a division that is also widely debated within the literature, particularly among humanitarian actors (2, 9–11). Respondents struggled to view humanitarian principles (neutrality, impartiality, and independence) and development cooperation principles (such as ownership, alignment, and mutual accountability) as compatible. The most common concern presented was how to be independent while working closely with national authorities and adhering to their rules and priorities (39, 40). However, we believe this argued conflict of principles is a false façade. Humanitarian principles are integral to the identity of humanitarian actors and crucial to legitimizing and constructing their professional operations. Therefore, they should not be viewed as straitjackets, but rather as values that guide them and set aspirations, subject to context-based interpretation and application, as reasoned by Lie (12). Hence, in light of the documented practices where humanitarians have managed to adapt and abide by development cooperation principles, as well as pragmatically respond to local contexts and authorities’ requirements, the divide can be bridged. The dialog between actors in these two domains within each country is the most effective way to configure this, maintaining and respecting the humanitarian space while adopting the nexus, in line with the reasoning of the International Council of Voluntary Agencies (34).

The claim that humanitarians only take up the nexus as an approach to get further grants was striking, yet not new. The New Humanitarian, part four of their “searching for the nexus” series, has reflected that “while some cash-strapped humanitarian relief agencies are hoping the nexus can unlock the generous budgets of development aid for crisis needs, it is not that simple” (41). They argue that the nexus presents challenges when attempting to extend humanitarian actions into new domains that humanitarian actors are unfamiliar with, and humanitarian work principles and policies are not consistent with, especially in the context of extensive political effects. This raises concerns about the statement that humanitarian funding is apolitical, which is difficult to substantiate when there is evidence that donors tend to avoid providing funding to countries with political gridlocks and unpredictable conditions (42).

The raised concerns about national systems and mechanisms (procurement, finance, information, and management) strengthening needs, responsibilities, and utilization problems require further studies to pave the way toward understanding the partners’ divide in Sudan. The claim that it does not matter which systems are to be used, a single nationally owned or parallel systems enrolled by international partners, is an extreme expression of nexus resistance, as it is commonly agreed that national systems are essential to sustain service provision and are the only way to phase out of humanitarian dependency. This is incorporated into the nexus definition for health within the WHO nexus guide (11). Meanwhile, finger-pointing and placing responsibilities on the other side hints at tensions between humanitarians and development partners that could threaten the implementation process of the nexus if not addressed. There is a need for a mindset change.

### 4.4 Methodological considerations and limitations

We acknowledge that, despite our attempts to triangulate and ensure strong contextualization, there are some limitations. The national viewpoint from Sudan was only presented through Government respondents, while no civil society actors were interviewed. We argue that, so far, civil society has not been involved in this nexus conceptualization and implementation process, and hence they were not critical respondents for this study. Key informant interviews were the main source of data, and although document review, coordination meeting minutes, and observation were utilized to recheck the data, they barely provided primary points to be presented, and rather enriched the discussion. The study focused on insights from health sector actors. Having non-health actors involved could have provided valuable insights into the process. Their perspectives could have highlighted different challenges, opportunities, and ways to improve the coordination, building on the health sector experience where some of them are engaged through the health sector partners forum (e.g., Ministries of Finance, social welfare, foreign affairs, etc.).

## 5 Conclusion

The study documented some partners’ efforts that aimed to apply the nexus approach for health systems strengthening, focusing on the sustainability of services by building on local capacities and system resilience against recurrent emergencies. However, the nexus suffered from a lack of clarity in its understanding. National ownership and embracing robust partners’ coordination benefited the process in Sudan. Apart from actors’ blame and claims against each other, the shared attempts on the nexus implementation, within this study, aided in arguing that the humanitarian and development divide can be bridged through dialog and respect. In such a volatile and challenging context, it is crucial to adopt a continuum approach to observing the nexus, entailing flexibility and adaptability in implementing the changing context. There is a clear need to intensify efforts to develop guidance and document best practices in implementing the approach, as well as to encourage further discussions and exchange of experiences between humanitarian and development partners across sectors, and to conduct additional cross-country case studies.

## Data Availability

The raw data supporting the conclusions of this article will be made available by the authors, without undue reservation.
