# Supplementary material for: Humanitarian–development nexus approach to health systems strengthening in Sudan—a policy analysis
Source: Front Public Health. 2025 Jun 18;13:1579825. doi: 10.3389/fpubh.2025.1579825 (PMC12213732; doi:10.3389/fpubh.2025.1579825)
Supplement: Supplementary file 1 [file Supplementary_file_1.docx]

Supplementary Material

# Interview Guide

***Interview No: …….***

**Introduction**

Thank you for accepting to join this study. The study aims to explore the understanding and conceptualization process of the humanitarian-development nexus approach to health system strengthening in Sudan.

**Checklist**

Interview duration: … minutes.

Interview place: ……………………

Request to record the interview: Approved or not

Explanation of how information will be handled with confidentiality: Explained or not

Request for permission to quote: Approved or not

Name of interviewee: ……………………

Interviewee agency: ……………………..

Function: ……………………

Date and time of interview: ……………………

**Nexus Understanding, introduction and conceptualization**

1. How do you perceive the concept of the nexus?
2. How did you get familiar with the nexus concept?

How was it introduced to the health sector? When and by who?

1. Can you describe your view on the status of partnership for health for both humanitarian and development partners?
2. How do you see the progress in the nexus use in Sudan health sector?

**The nexus in practice in Sudan health sector**

1. How does your organization/entity perceive and act on the nexus concept?

Has this been affected by any contextual factors? Kindly elaborate

1. Are there any players who lead and coordinate the operationalization of the NEXUS in Sudan? Has this changed overtime?
2. What are the changes or paradigm shifts necessary to enable the nexus approach? What can be the enablers and motives for these paradigm shifts?

Has this been affected by context changes?

1. Have your organization/entity embarked on adopting any nexus linked project(s)? kindly describe and elaborate any changes with context shifts in Sudan.
2. Are you aware of any practices and projects that are relevant to the nexus concept carried out by other partners? If yes, describe those projects and all known progress
3. Were there any platforms that influenced your conceptualization and action on the nexus? Which and how?

**Challenges and opportunities for the nexus implementation in Sudan health sector**

1. What are the main challenges you experience related to the implementation of the nexus and nexus inspired projects? (Explore the root causes behind those challenges)

What can be done to mitigate those challenges and the contextual factors?

1. What opportunities do you foresee to support the nexus implementation within Sudan health sector?

**Concluding remarks**

Is there anything you want to add to this interview?

Ask if the interviewee knows others who are important to interview. Why and if interviewee can facilitate the process?

Ask for any relevant documents

Thank the respondent
